# A novel mechanism of regulation of the anti-metastatic miR-31 by EMSY in breast cancer

**DOI:** 10.1186/s13058-014-0467-x

**Published:** 2014-11-18

**Authors:** Laoighse Mulrane, William M Gallagher, Darran P O’Connor

**Affiliations:** UCD School of Biomolecular and Biomedical Science, UCD Conway Institute, University College Dublin, Belfield, Dublin 4, Ireland

## Abstract

miR-31 is well known as an anti-metastatic microRNA (miRNA) in the context of breast cancer. However, to date the mechanism of regulation of this miRNA has yet to be elucidated. The recent publication by Viré *et al.* in Molecular Cell [1] demonstrates for the first time that one mechanism of regulation of miR-31 is through the putative oncogene EMSY, whose amplification in breast cancer patients correlates with reduced expression of the miRNA. This regulation is dependent on the DNA-binding transcription factor ETS-1 which recruits EMSY and the histone demethylase KDM5B to the miR-31 promoter, thus repressing its transcription.

**Electronic supplementary material:**

The online version of this article (doi:10.1186/s13058-014-0467-x) contains supplementary material, which is available to authorized users.

## Background

miRNAs are small non-coding RNA species which primarily negatively regulate gene expression, thus affecting a plethora of cancer-associated phenotypes. miR-31, a well-known anti-metastatic miRNA in breast cancer, was first shown to inhibit breast cancer metastasis both *in vitro* and *in vivo*[1] through the targeting of integrin-α5 (ITGA5), radixin (RDX) and RhoA [2] and later through targeting of WAVE3 [3]. Further investigation reported that suppression of ITGA5, RDX and RhoA recapitulated the phenotype produced by ectopic expression of miR-31 *in vitro* and *in vivo*[4]. Moreover, re-introduction of miR-31 expression in established xenograft MDA-MB-231 lung metastases was shown to result in metastatic regression, indicating that re-expression of miR-31 may be of therapeutic benefit in clinically advanced patients [5].

EMSY, a putative oncogene involved in gene regulation, is known to be amplified in approximately 13% of sporadic breast cancers [6]. However, the locus in which this gene is contained (11q13-14) includes a number of other genes implicated in breast cancer including CCND1, PAK1, CTTN, and FGF3, making it difficult to decipher the exact contribution of EMSY to the poor prognosis associated with amplification of this region. A recent article in Molecular Cell has gone some way to elucidating the function of this gene in breast cancer, describing a role for EMSY in oncogenic transformation as well as invasion/migration in breast cancer through the regulation of miR-31 [7].

## Article

**Figure 1:**
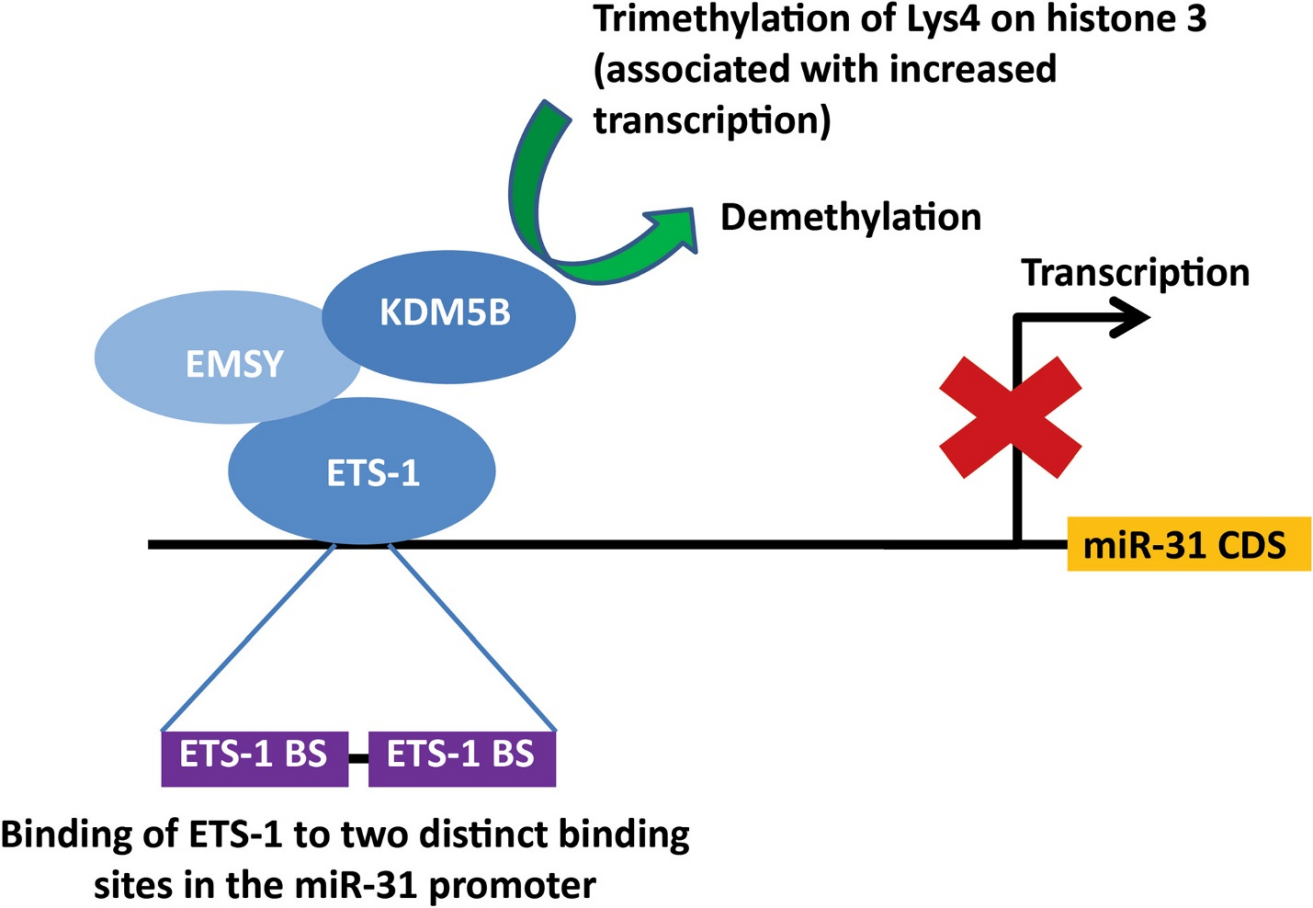
**Proposed mechanism of regulation of miR-31 in breast cancer (adapted from** **[**[7]**]).** ETS-1 recruits oncogene EMSY and H3K4me3 demethylase KDM5B to the promoter of miR-31, repressing transcription of the pri-miRNA.

The publication from the Kouzarides group reports that the oncogene EMSY targets miR-31 through co-operation with the transcription factor ETS-1 and the histone lysine demethylase KDM5B, resulting in transcriptional repression of the miRNA and numerous phenotypic effects (Figure 1).

Firstly, the group investigated the effect of modified expression of EMSY in cell lines and xenograft models. Ectopic expression of the oncogene in MCF7 cells resulted in increased growth in an orthotopic mammary fat pad xenograft model while an experimental metastasis tail vein injection model of the same cells demonstated increased lung micrometastases relative to the control line. Given the potential role for this gene in transcriptional regulation, a qRT-PCR-based screen was employed to profile 88 miRNAs in MCF7 cells in which EMSY had been depleted revealing 38 to be altered in this context. A second approach was then utilised whereby re-analysis of the METABRIC cohort [8],[9] for miRNAs altered in EMSY-amplified (cis-acting aberrations of the 11q13-14 locus) versus non-amplified cases revealed dysregulation of 12 miRNAs. As miR-31 was the only miRNA to be identified using both approaches, it was chosen for further studies.

miR-31 expression levels inversely correlated with EMSY levels in patient samples from the METABRIC dataset and in cell lines with amplification or ectopic expression of EMSY. Finding that expression of EMSY increased both oncogenic transformation and migration *in vitro*, the Kouzarides group then demonstrated that re-expression of miR-31 had the ability to abrogate both phenotypic effects of EMSY oncogenicity, while modification of the expression of the miRNA alone altered invasion and migration and phenocopied the effects of EMSY depletion.

The authors then investigated the mechanism of miR-31 repression by EMSY demonstrating recruitment of EMSY to the miR-31 promoter, thus repressing transcription of the primary miRNA (pri-miRNA). However, as EMSY contains no DNA-binding domain another factor was necessary for promoter recruitment. Bioinformatic analysis of the miR-31 promoter revealed binding sites for ETS-1, ETV4/PEA3 and GATA1 with site-directed mutagenesis of any of the ETS-1 binding sites in the promoter reducing promoter activation. ETS-1 was subsequently confirmed to bind to the miR-31 promoter. Furthermore, this transcription factor was then shown to recruit the histone H3K4me3 demethylase KDM5B to the miR-31 promotor, thus inhibiting transcription of miR-31 through the demethylation of Lys4 on histone 3.

## Viewpoint

This article is the first to mechanistically anchor an oncogenic role for EMSY and demonstrate a direct mechanism of regulation of miR-31, an important metastasis-associated miRNA, in breast cancer. Numerous mechanisms of miRNA regulation exist, including epigenetic silencing via DNA methylation or histone modification [10], direct regulation by nuclear receptors [11] or indirect regulation through alteration of the miRNA biogenesis pathway [12]. miR-31 has been seen to be hypermethylated in some cell lines [13] and a recent study demonstrated that the miR-31 promoter also undergoes aberrant methylation in breast cancer patients [14]. However until now, no direct regulatory mechanisms have been described.

The evidence presented from both *in vitro* and *in vivo* studies provides a convincing argument for the classification as EMSY as an oncogene, as well as the direct regulation of miR-31 by this oncogene. However, the story is undoubtedly much more complex, as EMSY may regulate a number of different miRNAs/genes. Nor is this the only potential mechanism of direct regulation of miR-31. Notably, as EMSY amplification is only seen in approximately 13% of sporadic breast cancers, it is imperative to also study the regulation of this miRNA in an EMSY non-amplified context. Moreover, given that there were only 45 samples with EMSY amplification and corresponding miRNA data in the cohort studied, expanding the analysis into larger cohorts would provide additional evidence as to the relevance of this discovery in breast cancer patients.

Viré and colleagues have elegantly documented a novel role for EMSY in breast cancer. However, they have also presented the case for a second scenario, that in which the oncogenic properties of this gene may be functionally propagated by potential oncomiRs. Indeed, EMSY depletion in cell lines led to the upregulation of 29 miRNAs and 6 miRNAs were found to be overexpressed in breast cancer patients with amplification of the gene. The mechanism of targeting of these miRNAs by EMSY, either directly or indirectly, has yet to be investigated.

To date, the focus of the majority of miRNA publications has been to evaluate gene targets observed to affect a functional response. This study highlights a need to fully elucidate the mechanisms of regulation of the miRNAs themselves, not just their targets. This may lead to the discovery of a myriad of feedback loops similar to that between the miR-200 family of miRNAs and the transcriptional repressors ZEB1/SIP1 which functions to regulate epithelial-mesenchymal transition (EMT) [15]. As miRNA inhibitors/mimics have only recently begun to enter clinical trials, ascertaining the mechanism of regulation of key miRNAs may greatly improve the chances of finding a surrogate gene candidate which could be successfully targeted in the clinic.

## Authors’ original submitted files for images

Below are the links to the authors’ original submitted files for images. Authors’ original file for figure 1

## Abbreviations

EMT: epithelial-mesenchymal transition
miRNA: microRNA
pri-miRNA: primary microRNA
