# Supplementary figures and images for: A novel mechanism of regulation of the anti-metastatic miR-31 by EMSY in breast cancer
Source: Breast Cancer Res. 2014 Nov 18;16:467. doi: 10.1186/s13058-014-0467-x (PMC4429417; doi:10.1186/s13058-014-0467-x)

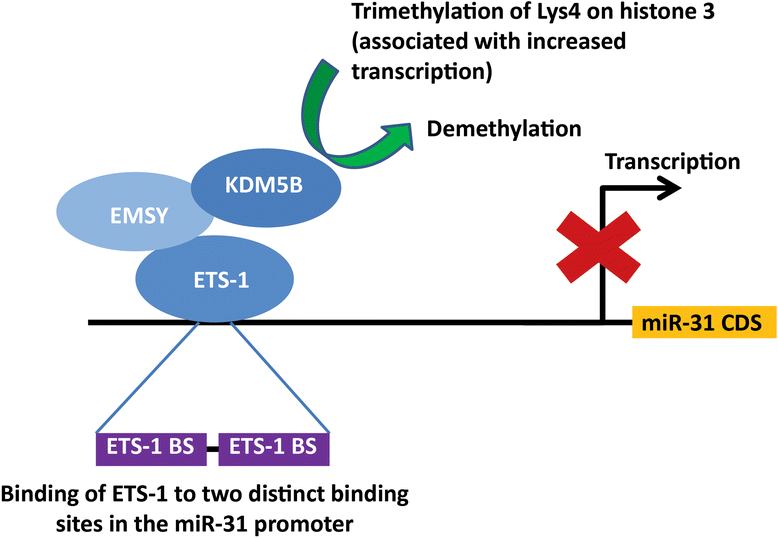

Supplement: Supplementary file 1 — Authors’ original file for figure 1 [file 13058_2014_467_MOESM1_ESM.gif]
